# Greater Perceived Age Discrimination in England than the United States: Results from HRS and ELSA

**DOI:** 10.1093/geronb/gbv040

**Published:** 2015-07-29

**Authors:** Isla Rippon, Paola Zaninotto, Andrew Steptoe

**Affiliations:** Department of Epidemiology and Public Health, University College London, UK.

**Keywords:** Age discrimination, Ageism, Cross-National Studies, Older adults

## Abstract

**Objectives.:**

We examined cross-national differences in perceptions of age discrimination in England and the United States. Under the premise that the United States has had age discrimination legislation in place for considerably longer than England, we hypothesized that perceptions of age discrimination would be lower in the United States.

**Methods.:**

We analyzed data from two nationally representative studies of aging, the U.S. Health and Retirement Study (*n* = 4,818) and the English Longitudinal Study of Ageing (*n* = 7,478). Respondents aged 52 years and older who attributed any experiences of discrimination to their age were treated as cases of perceived age discrimination. We used multivariable logistic regression to estimate the odds ratios of experiencing perceived age discrimination in relation to selected sociodemographic factors.

**Results.:**

Perceptions of age discrimination were significantly higher in England than the United States, with 34.8% of men and women in England reporting age discrimination compared with 29.1% in the United States. Associations between perceived age discrimination and older age and lower levels of household wealth were observed in both countries, but we found differences between England and the United States in the relationship between perceived age discrimination and education.

**Discussion.:**

Our study revealed that levels of perceived age discrimination are lower in the United States than England and are less socially patterned. This suggests that differing social and political circumstances in the two countries may have an important role to play.

Discrimination whether it is based on age, sex, race, or other characteristics can be regarded as unfair treatment. Perceived discrimination can be defined as an individual’s perception of being treated unfairly by others due to a personal attribute, such as, age, gender, or race (Ayalon & Gum, 2011; Kessler, Mickelson, & Williams, 1999). Where age discrimination can be argued to differ from other forms of discrimination is that we are all at risk of experiencing it at some point in our lives (Gee, Pavalko, & Long, 2007). The term age discrimination is often linked with, or regarded as an element of, the term ageism, a term that was first introduced by Robert Butler in 1969. He regarded it as the “disease” which leads to discrimination and prejudice against one age group by another (Butler, 1969). He identified three interrelated aspects of ageism: prejudicial attitudes towards older people; discriminatory practices against older individuals, for example, in employment and other social settings; and institutional practices and policies which can perpetuate certain stereotypes about older age (Butler, 1980; Wilkinson & Ferraro, 2002). All of these may impact on an older person’s quality of life. Equally important is the individual’s perception that he or she experiences discrimination on the basis of their age. The extent to which this perception reflects real occurrences of discriminatory attitudes or behaviors of other people or institutions is often difficult to trace, but it can be argued that perceptions are what matter in this context as they do in many other socially prescribed situations (Schmitt, Branscombe, Postmes, & Garcia, 2014). In this article, the term age discrimination is used to describe any experiences where an individual feels they have been treated in an unfair or in a different way due to their age.

Previous research has shown that perceived discrimination in everyday situations is associated with physical as well as mental health status and wellbeing (Ayalon & Gum, 2011; Kessler et al., 1999; Luo, Xu, Granberg, & Wentworth, 2012; Pascoe & Smart Richman, 2009; Pavalko, Mossakowski, & Hamilton, 2003). Perceived discrimination may act like a stressor that can build up over time, eventually taking a toll on an individual’s mental and physical health and wellbeing (Kessler et al., 1999). Another implication of this is that frequent exposure to perceived age discrimination could lead to social withdrawal and a reduction in cultural engagement in order to avoid potential discriminatory situations.

While ageism may not have received the same level attention as sexism or racism in the past, it is of growing importance. As the proportion of older adults in both the United States and England increases, these changes to population structures will have important economic as well as social implications. As a result, the effects of ageism will need to be identified and better understood. The United States and England have differing legislative environments and attitudes to age, so the comparison is interesting. In both the United States and England, debates around age discrimination in the workplace have existed since the 1930s (Macnicol, 2006). In the United States, legislation to end age discrimination in the workplace has been in place for over 45 years, with successive amendments bringing mandatory retirement age to a virtual end (ILC-USA Anti-Ageism Task Force, 2006). By contrast, England has only relatively recently passed legislation on age discrimination. First, through 2006 employment legislation, and subsequently through the broader Equality Act 2010, age discrimination legislation was extended to cover the provision of services and public functions. Previous research has shown that U.S. age discrimination legislation has had a positive impact on employment through the retention of older workers, but that it has not been as effective for those seeking work (Lahey, 2010; Lain, 2011). However, it is notable that there has been less discussion in the United States around extending age discrimination legislation to cover services as has been seen in England (Macnicol, 2012).

Although the implementation of legislation has been of importance, it does not directly cover the experiences of discrimination that may occur on a frequent or daily basis—the personal attacks on an individual’s character. In addition to the legislative environment, age discrimination may arise through prejudicial attitudes towards older persons, and the prevalence of stereotypes about older people. Because of the subjective nature of perceived age discrimination, the culture of the two countries may influence these perceptions and the age-related attitudes that may result. At the individual level, Levy argues that stereotypes of ageing are embodied when their assimilation from the surrounding culture leads to self-definitions that in turn influence functioning and health (Levy, 2009). As these age-related stereotypes are assimilated over the life course, they may gain relevance at older ages and affect actual ageing experiences almost unknowingly, including many health outcomes. However, evidence from a recent empirical study suggests that these age-related stereotypes have the potential to be changed so that interventions may be possible (Levy, Pilver, Chung & Slade, 2014).

Comparisons between countries may throw light on the drivers of discrimination and may offer insights into perceptions of discrimination. Various studies have been carried out in the two countries on attitudes towards older adults and on discriminatory experiences, but less on individual perceptions of discrimination. However, the focus of much research on perceived discrimination in the United States in particular has been on racism or incidence of discrimination more broadly (Ayalon & Gum, 2011; Kessler et al., 1999; Williams, Neighbors, & Jackson, 2003; Williams, Yan, Jackson, & Anderson, 1997). While a number of studies have begun to focus on age discrimination there are still relatively few which have used large representative samples of older adults. Previous research using weighted data from English Longitudinal Study of Ageing (ELSA) found that around one-third of over 52 year olds in England reported perceptions of age discrimination (Rippon, Kneale, de Oliveira, Demakakos, & Steptoe, 2014). Studies using data from the Health and Retirement Study (HRS) and Midlife in the United States (MIDUS) surveys in the United States have found that approximately 30% of over 65 year olds gave age as the reason for their discriminatory experience (Ayalon & Gum, 2011; Kessler et al., 1999; Luo et al., 2012). Analyses of the 2008 European Social Survey showed that on average 26% of European citizens over 62 year olds have experienced discrimination due to their age, with over 1 in 10 frequently experiencing discrimination (Age England; Van den Heuvel & van Santvoort, 2011). However, one difficulty in making international comparisons is that different measures have often been used. ELSA and HRS offer a way to address this, as the two studies have been developed in a complementary fashion so as to facilitate cross-national comparisons through use of identical measures (Steptoe, Breeze, Banks, & Nazroo, 2013).

Existing studies indicate that besides age, experiences of greater age discrimination have variously been found to be associated with women, lack of paid employment, not being married, ethnicity, fewer years of education, and lower socioeconomic status (SES) as defined by household income or occupational social class (Abrams, Vauclair, & Swift, 2011; Luo et al., 2012; Sweiry & Willitts, 2012; Van den Heuvel & van Santvoort, 2011; Yuan, 2007). However, the strength and direction of these associations has differed across studies. For example, studies using data from the European Union have indicated that women are more likely to experience discrimination than men, while research using data from the United States has shown that men perceive higher levels of day-to-day discrimination than women (Demos, 2012; Kessler et al, 1999; Sweiry & Willitts, 2012).

To our knowledge, few studies have used large scale nationally representative data to analyze perceived age discrimination in older adults or to evaluate potential cross national differences in discrimination between these two countries. In this article, we focus on cross-national differences in perceptions of age discrimination in the United States and England, and the extent to which older adults in both countries attribute experiences of discrimination in their day-to-day lives to their age. Our aims are, firstly, to investigate whether or not there are differences in the overall levels of perceived age discrimination reported by older adults in the United States and England; and secondly, to examine the sociodemographic correlates of perceived age discrimination in the two countries. Our final aim is to investigate whether these results differ across individual discriminatory situations. We hypothesized that perceived age discrimination would be lower in the United States in comparison with England as age discrimination legislation has been in place for a longer period and that there would be a greater awareness of it in England due to the recent discourse around age discrimination before and after the legislation’s implementation. Therefore, assimilation of cultural and institutional attitudes to age may influence respondents’ perceptions of the level of age discrimination in the two countries. Second, based on previous research we expected that sociodemographic correlates would be associated with perceived age discrimination in a similar fashion. Therefore we included a number of the key sociodemographic characteristics identified in previous studies from the United States and Europe that have been shown to be associated with perceived discrimination in order to test this assumption. Although we expected overall levels to differ across countries, we expected that among those individuals who attributed a perceived incidence of discrimination to their age, social characteristics such as wealth, education, older age, and work status would be predictors of perceived discrimination in both countries. In regard to the individual discriminatory situations, we hypothesized that perceived age discrimination would be lower in the United States in the majority of the five specific situations evaluated, but not in relation to health care. Due to the greater inequality in access to health care in the United States in comparison with England (Davis, Stremikis, Squires, & Schoen, 2014), we predicted that perceived age discrimination would be higher in this situation in the United States.

## Methods

### Study Samples

The samples were drawn from two longitudinal studies of aging, the U.S. HRS and ELSA; the two surveys were developed collaboratively with significant overlap in the questions in order to facilitate cross-national comparisons. To maximize comparability between the two study populations, we included people aged 52 and older only and restricted the sample to non-Hispanic White respondents only due to the very low numbers of non-White respondents in ELSA (282 respondents or ~3% of the core sample).

We used data from Wave10 (2010) of HRS to assess age discrimination in the United States. HRS is longitudinal study of over 50 year olds which commenced in 1992 (National Institute on Aging, 2007; Sonnega et al., 2014). The sample was selected using a multistage area probability sample design, with oversamples of African-Americans, Hispanics, and residents of Florida and is refreshed periodically. The response rate for the main interview in 2010 was 88.6%. Since 2006 the study has included a self-completion questionnaire collecting data on psychosocial measures. The perceived discrimination measures were included within this leave-behind questionnaire which is sent randomly to approximately half of the HRS participants at each wave (Smith et al., 2013). After exclusion of 44 respondents aged 51 years or younger and 899 non-Hispanic White respondents, a total of 4,822 participants responded to the discrimination questions in 2010. Data were missing on one or more covariates for eight individuals, giving a final HRS sample of 4,818 respondents.

For England, data were drawn from Wave 5 (2010–11) of ELSA which was the first wave to include the measures of perceived discrimination. ELSA is a longitudinal panel survey of aging and quality of life among men and women aged 52 and older living in private households in England, which commenced in 2002–03 (Steptoe et al., 2013). The initial sample was selected from three survey years of the Health Survey for England (1998, 1999, and 2001)—an annual government health survey based on a stratified random sample of all households in England. Households were included if they contained at least one individual who was aged 50 or older and who had agreed to be contacted again in the future. The ELSA sample is reassessed every 2 years and is periodically refreshed to ensure a representation of younger participants. Data are collected each wave using computer-assisted personal interviews (CAPI), and self-completion questionnaires, and from a nurse visit every 4 years (Waves 2, 4, and 6). The mean cross-sectional response rate for Wave 5 was 80.1% (Banks, Nazroo, & Steptoe, 2012). Among the 9,090 core participants who were interviewed at wave 5 of ELSA, 8,003 non-Hispanic Whites answered the self-completion questionnaire that contained the measures of age discrimination. A further 302 had missing responses to the discrimination questions. Data were missing on one or more covariates for 149 individuals, primarily wealth. The analytic sample therefore comprised 7,478 participants.

### Measures

#### Perceived Age Discrimination

Both HRS and ELSA have included questions on perceived everyday discrimination in their self-completion questionnaires. Respondents were asked about the frequency of five discriminatory situations as follows: “In your day-to-day life, how often have any of the following things happened to you?”

1. You are treated with less respect or courtesy than other people
2. You receive poorer service than other people in restaurants and stores
3. People act as if they think you are not clever (ELSA)/smart (HRS)
4. You are threatened or harassed
5. You receive poorer service or treatment than other people from doctors or hospitals

Possible response options ranged from 1 (Almost every day) to 6 (Never). As the data were skewed, with most participants reporting discrimination less than once a year or never in any of the discriminatory situations, we dichotomized the responses to indicate whether or not participants had experienced discrimination in the past year (a few times or more a year vs. less than once a year or never), with the exception of the fifth item which was dichotomized to indicate whether or not respondents had ever experienced discrimination from doctors or hospitals (never vs. all other options). A follow-up question asked respondents to indicate what reason/s they attributed their experience in any of the five discriminatory situations. Possible options included: age, gender, race, weight and physical disability, and participants were able to select more than one reason. Participants who attributed any experiences of discrimination to their age were treated in our study as cases of perceived age discrimination.

### Covariates

Our analyses took six sociodemographic measures into account: age, sex, wealth, education, marital status, and current work status. Age was split into four categories for the purpose of analysis: 52–59 years, 60–69 years, 70–79 years, and a final group combining all those aged 80 and older. Sex was coded 1 for female and 0 for male. Two measures of SES were included: wealth and education. Total household wealth (excluding pensions or individual retirement accounts) was divided into country-specific equally sized wealth quintiles. Wealth is regarded as the best indicator of socioeconomic resources at older ages (Banks, Karlsen, & Oldfield, 2003). In the case of education, American education was divided into low (less than high school), intermediate (high school graduate), and high (some college through to college graduate or more). English education was measured by the highest educational qualification attained and divided into three groups: low (qualifications below O-Level or no educational qualification), intermediate (A-Levels, O-Levels, or equivalent), and high (those with higher education below a degree through to higher degrees). Marital status was coded into four categories: married or remarried, single, separated or divorced, and widowed. Finally current work status indicated whether or not a respondent was currently employed, retired or in another situation, for example, unemployed or looking after the home or family.

### Statistical Analyses

The primary outcome was the perception of age discrimination in any of the five discriminatory situations in the United States and England. The secondary outcomes were perceptions of age discrimination in each of the five individual discriminatory situations. We analyzed the data in five main steps. Firstly, we used chi-square tests to assess the bivariate relationships between perceived age discrimination and individual covariates in both the United States and England. Secondly, we conducted multivariable logistic regression analysis for each country separately, with perceived age discrimination as the dependent variable, adjusting for all covariates. Next, the data from the HRS and ELSA samples were then pooled, and a dummy variable indicating country was included in the regression model in order to determine any cross-national differences in age discrimination. To further examine potential country differences, we ran a series of logistic regression models including interaction terms in order to examine whether the associations between sociodemographic characteristics and perceived age discrimination differed significantly between the countries. Lastly, to test our final hypothesis we analyzed the individual discriminatory situations in five separate models in order to determine whether country effects were the same across the different situations. The outcome variable in each of these five models was the proportion of respondents who attributed an experience of discrimination to their age (e.g., respondents who perceived they had been treated with less courtesy in a situation and attributed this to their age). Statistical analyses were conducted using version 12.1 of Stata (Stata Corp, College Station, TX). The data were unweighted since the study combined two subsamples of respondents in the HRS and ELSA which had different weights. A previous study using weighted ELSA data produced similar results to this study (Rippon et al., 2014).

In addition, we conducted a sensitivity analysis using the data that included non-White respondents. These analyses showed similar patterns of the effects of perceived age discrimination as the sample which excluded non-White respondents.

## Results

Table 1 describes the sociodemographic characteristics of the two study populations. There were significant differences between the two countries for all sociodemographic characteristics with the exception of wealth. The U.S. cohort has a higher proportion of over 70 year olds, retired, well-educated, and widowed respondents in comparison with the English sample (all *p* < .001). The mean age of the HRS sample is 71.1 years old and the mean age of the ELSA sample is 67.4.

**Table 1.** Sample Characteristics by Country, and Bivariate Associations Between Age Discrimination and Sociodemographic Factors

**Variable**	**United States (%**)	**England (%**)	**Age discrimination United States (%) England (%**)		***p* value** ^**a**^
Total	4,818	7,478	29.1	34.8	<.001
Age in years					
52–59	12.9	21.4	26.7	27.6	.638
60–69	31.6	40.4	27.4	36.2	<.001
70–79	35.5	27.4	30.3	38.3	<.001
Over 80	19.9	10.8	31.3	35.2	.085
Sex					
Male	42.8	44.5	28.8	36.2	<.001
Female	57.2	55.5	29.3	33.8	<.001
Wealth					
Lowest 1	13.0	16.1	32.5	37.0	.054
2	16.8	19.8	32.3	36.9	.030
3	20.7	20.3	30.3	35.1	.012
4	24.0	21.4	27.3	34.4	<.001
Highest 5	25.5	22.3	26.0	31.6	.001
Education					
Low	17.5	24.6	30.6	31.7	.584
Intermediate	33.3	39.9	27.0	35.5	<.001
High	49.2	35.5	30.0	36.3	<.001
Marital status					
Married	69.0	66.4	28.0	34.5	<.001
Single	2.3	5.9	31.2	34.3	.537
Divorced or separated	8.8	11.9	31.5	35.8	.129
Widowed	20.0	15.9	31.7	35.7	.052
Work status					
Retired	73.5	60.2	29.9	38.0	<.001
Employed	20.4	28.5	25.8	29.5	.034
Other	6.1	11.3	30.5	31.6	.720

*Notes:* ^a^Chi-square test for differences between the United States and England.

Overall we found that perceived age discrimination was higher in England than the United States (Table 1). 29.1% of over 52 year olds in the United States reported age discrimination in comparison with 34.8% in England (*p* < .001), with this figure rising to 30.2 and 37.5% for over 70 year olds in the United States and England, respectively. A significantly higher proportion of individuals who were married, higher educated, retired, older, and across all wealth levels and of both sexes reported age discrimination in England than the United States.

We ran logistic regression models for the two countries separately (Table 2). The fully adjusted analyses revealed that perceived age discrimination was significantly associated with older age groups, higher levels of education, being retired, and lower levels of household wealth in the English sample. In the U.S. sample, respondents who perceived age discrimination were more likely to be older and to have lower levels of household wealth.

**Table 2.** Adjusted Odds Ratios of Reporting Age Discrimination by Country

	United States		England		Interaction
	OR (95% CI)	*p* value	OR (95% CI)	*p* value	*p* value
Age					
52–59	1.00		1.00		
60–69	1.03 (0.82–1.29)	.809	1.36 (1.17–1.58)	<.001	.040
70–79	1.16 (0.91–1.48)	.223	1.42 (1.19–1.70)	<.001	.184
Over 80	1.18 (0.90–1.54)	.239	1.24 (0.99–1.55)	.059	.760
Country × age^b^					.029
Sex					
Male	1.00		1.00		
Female	1.00 (0.89–1.15)	.949	0.92 (0.83–1.01)	.093	.288
Country × sex^b^					.148
Wealth					
1 (lowest)	1.00		1.00		
2	1.01 (0.81–1.27)	.921	0.96 (0.81–1.13)	.601	.698
3	0.92 (0.74–1.15)	.456	0.83 (0.70–0.98)	.029	.473
4	0.78 (0.62–0.97)	.026	0.77 (0.65–0.92)	.003	.960
5 (highest)	0.71 (0.57–0.89)	.003	0.66 (0.55–0.79)	<.001	.609
Country × wealth^**b**^					.798
Education					
Low	1.00		1.00		
Intermediate	0.90 (0.75–1.08)	.263	1.34 (1.18–1.53)	<.001	.001
High	1.14 (0.95–1.37)	.152	1.52 (1.32–1.76)	<.001	.014
Country × education^b^					.002
Marital status					
Married	1.00		1.00		
Single	1.08 (0.72–1.27)	.703	0.98 (0.79–1.21)	.840	.666
Separated	1.10 (0.88–1.39)	.396	1.02 (0.87–1.20)	.792	.584
Widowed	1.08 (0.91–1.29)	.367	0.96 (0.83–1.12)	.630	.318
Country × marital status^b^					.393
Work status					
Retired	1.00		1.00		
Employed	0.86 (0.71–1.04)	.111	0.74 (0.64–0.85)	<.001	.239
Other	1.04 (0.80–1.35)	.785	0.82 (0.70–0.97)	.023	.147
Country × work status^b^					.097

*Notes*: CI = confidence interval.

^a^Model adjusted for country, age, sex, education, wealth, marital status, and work status.

^b^ *p* value of likelihood ratio test for an interaction between country and a sociodemographic variable.

To test differences between the two countries, the data were pooled. Using perceived age discrimination as the dependent variable, the fully adjusted logistic regression model showed that English respondents were significantly (OR 1.39; 1.28–1.51; *p* < .001) more likely to report age discrimination than the Americans (Table 3). Overall significant interactions of country with age and education, were found but not for wealth or gender (Table 2). Marked differences between the two countries were observed at the 60–69 age groups (*p* = .040) and a significant difference was observed between the two countries at both the intermediate (*p* < .001) and higher education categories (*p* = .014). Thus, the likelihood of perceiving age discrimination was significantly higher for English respondents aged 60–69 and in intermediate or higher education in comparison with their American counterparts.

**Table 3.** Unadjusted and Adjusted Odds Ratios from Logistic Regression of Reporting Discrimination in Different Situations and Attributing it to Age

	Undjusted OR (95% CI)	*p* value	Adjusted OR (95% CI)^a^	*p* value
Overall				
United States	1.00		1.00	
England	1.30 (1.20–1.41)	<.001	1.39 (1.28–1.51)	<.001
Treated with less courtesy than others				
United States	1.00		1.00	
England	1.28 (1.16–1.41)	<.001	1.22 (1.10–1.36)	<.001
Received poorer service or treatment than other people from doctors or hospitals				
United States	1.00		1.00	
England	1.13 (1.00–1.28)	.050	1.16 (1.02–1.31)	.026
People act as if they think you are not clever or smart				
United States	1.00		1.00	
England	0.85 (0.76–0.94)	.003	0.82 (0.73–0.92)	.001
Received poorer service than others in a restaurant or shop				
United States	1.00		1.00	
England	1.13 (0.99–1.29)	.062	1.09 (0.95–1.25)	.218
You are threatened or harassed				
United States	1.00		1.00	
England	1.72 (1.40–2.11)	<.001	1.55 (1.25–1.91)	<.001

^a^Model adjusted for country, age, sex, education, wealth, marital status, and work status.

For each of the five individual discriminatory situations, the proportion of respondents who perceived discrimination in a particular situation and attributed it to their age was calculated. The prevalence of respondents reporting perceived age discrimination ranged from 18.2% and 14.8% in England and the United States, respectively, for those who were treated with less courtesy to 2.7% and 4.5% for those who experienced harassment (both *p* < .001). Americans reported higher rates of age discrimination in only one of the five discriminatory situations; 12.9% of American respondents thought that they were treated as less smart because of their age, compared with 11.1% of English respondents (*p =* .003). 9.2% of Americans and 10.3% of English participants attributed the occurrence of discrimination in medical settings to their age (*p* = .05) (Table 3). Similar results were found in the adjusted model, where we also found very little or no difference between the two countries regarding age discrimination experienced in service settings (Table 3). In the situations where people perceived they were treated with less courtesy or were harassed, a higher proportion of individuals who were married, higher educated, retired, older and across all wealth levels and male reported age discrimination in England in comparison with the United States. The reverse was the case where individuals perceived they were treated as less smart (see Supplementary Table 1). In service and medical settings, very few differences were observed between the two countries.

## Discussion

This study compared levels of perceived age discrimination in the United States and England using nationally representative samples of older adults. Using the same measure of perceived discrimination, our results indicate that perceptions of age discrimination are higher in England than the United States, with 34.8% of men and women aged 52 years and older in England reporting age discrimination compared with 29.1% in the United States. In the fully adjusted multivariate model, English participants were significantly more likely to report age discrimination (OR 1.39; 1.28–1.51; *p* < .001). It is possible that older men and women in the U.S. encounter less age discrimination than their English counterparts, so fewer perceive age discrimination. But an alternative explanation for the higher levels of age discrimination in England is that English respondents are more aware of age discrimination and therefore more readily report it, or are more likely to label an experience as due to age discrimination. Equally this may provide evidence of the role that surrounding culture may play in the development of self-stereotypes of aging and in turn influence individuals’ perception of age discrimination in the two countries. The more recent introduction of legislation and the resulting discourse around it may have sensitised individuals to age discrimination more strongly in England in comparison with the United States where such legislation has been in place for over 45 years (Abrams & Swift, 2012). Further, it has been argued that despite evidence of age discrimination and how it affects quality of life, many Americans perceive it as less serious than other forms of discrimination, such as, racism and sexism (ILC-USA Anti-Ageism Task Force, 2006).

The second objective of our study was to investigate sociodemographic characteristics associated with perceived age discrimination in the United States and England. Our findings also indicate that there were some important differences between the countries in the correlates of age discrimination, and suggest that our second hypothesis was too broad since the relationships between perceived age discrimination and age, education, marital status, and work status all differed. In the U.S. sample, perceived age discrimination was more common in older age groups and people with less wealth. In the English sample, perceived age discrimination was also more common in older and less affluent respondents, but in addition it was associated with higher levels of education and being retired. This could suggest that perceptions of age discrimination in older age groups are less socially patterned in the United States than England.

In agreement with previous studies, we observed an inverse gradient between perceived age discrimination and SES, in this instance indexed by wealth, with individuals in the lowest wealth quintile more likely to experience age discrimination than wealthier respondents in both countries (Kessler et al., 1999; Lee & Turney, 2012; Luo et al., 2012; Sweiry & Willitts, 2012; Van den Heuvel & van Santvoort, 2011; Yuan, 2007). Thus, the proportion of respondents reporting perceived age discrimination rose from 26.0% and 31.6% in the wealthiest U.S. and English quintiles to 32.5% and 37% in the least wealthy quintiles. Wealth potentially protects individuals from exposure to situations that give rise to discrimination and provides a greater sense of control or security. We found contrasting results for the relationship between perceived age discrimination and level of education in the two countries. In the HRS sample, no association was observed between age discrimination and education, but a positive association was observed in ELSA, where respondents with higher levels of education were more likely to report age discrimination. While it would have been expected that the two measures of SES would follow an inverse gradient, some studies using data from the United States and Europe have reported no significant associations between education and everyday discrimination, (Ayalon & Gum, 2011; Kessler et al., 1999; Luo et al., 2012), while others have reported a positive association between education and discrimination (Gee et al., 2007; Van Den Heuvel & van Santvoort, 2013). A previous study using data from ELSA also showed a positive association between education and age discrimination, despite using different groupings of education for England (Rippon et al., 2014). The unexpected association between high education and greater perceived age discrimination in England but not in the United States could highlight cultural differences between the two countries, or reflect differences within the education systems in both countries.

Retired respondents in England were more likely to report perceived age discrimination than those who were employed. This is consistent with analyses of other data from the England (Abrams, Eilola, & Swift, 2009). In the U.S. sample, no significant relationship between work status and age discrimination was observed, suggesting that there is less of a marked transition between work and retirement in the United States. This may reflect the effective abolition of mandatory retirement in the United States several decades ago, while this occurred in England only in 2006. However, in our study it is hard to establish whether legislation has an impact on age discrimination in day-to-day situations.

The workplace is also an important context for older people to meet and interact with those of younger ages and could offer an explanation as to why those who are retired perceived greater age discrimination in comparison to those in work (Abrams & Swift, 2012). Previous research on prejudice and discrimination has tended to argue that increasing the quality of contact between different social groups, in this instance, between younger and older generations, is the best intervention to reduce discrimination (Richeson & Shelton, 2006). Stereotypes of older age are argued to reflect the lack of contact between different generations.

Women perceived less age discrimination than men in both countries, a finding that has been previously reported in relation to both every day and major incidents of discrimination (Jang, Chiriboga, & Small, 2008; Kessler et al., 1999; Lee & Turney, 2012; Luo et al., 2012). It has been argued previously that women are more likely to deny or discount experiences of discrimination which may lead to underestimation (Crosby, 1984; Kessler et al., 1999). While women may report less discrimination, it has also been found that everyday discrimination is more strongly associated with poorer mental health in women while major discriminatory events are more strongly associated with mental health in men (Lee & Turney, 2012). Equally, while we found that women perceived less age discrimination, it is likely that women are more likely to experience ‘double discrimination’ whereby they may perceive discrimination both due to their age and gender (Arber & Ginn, 1995).

Finally, we looked in detail at several individual discriminatory situations. Our findings revealed that in both countries age discrimination was perceived most where people were treated with less courtesy and least where people experienced actual harassment. In both instances, rates were higher in England in comparison with the United States. Overall, we observed virtually no difference between the countries regarding perceived age discrimination in service settings. It has been shown previously that older adults may encounter patronizing communication when interacting with strangers in public places such as shops or restaurants and that negative ageist stereotypes may explain or reinforce such reactions (Kite, Stockdale, Whitley, & Johnson, 2005; Nussbaum, Pitts, Huber, Krieger, & Ohs, 2005).

Contrary to our prediction we found that approximately 10% of the sample in both countries reported perceived age discrimination in a hospital or from a doctor. We had expected that the disparities in health care access in the United States might lead to greater perceived discrimination (Davis et al., 2014), but this was not the case. Nevertheless, our findings provide further evidence of the existence of age discrimination in medical settings, an area that previous research has identified as a particular problem (Braithwaite, 2002; Greene, Adelman, Charon, & Hoffman, 1986). Age discrimination may be evident in how clinical staff communicate or interact with older patients and in the quality of care older patients receive in comparison with younger patients (Nussbaum et al., 2005; Pasupathi & Lockenhoff, 2002).

One of the main strengths of this study is that we used data from two nationally representative cohorts of over 50 year olds in England and the United States. However, there are several limitations and caution is needed when interpreting these findings. Firstly, it is not possible to establish causal relationships in this cross-sectional study. We do not know whether older people are more likely to experience discrimination because of their age or whether they are more likely to attribute discrimination to age as they get older. Longitudinal data would enable us to see whether rates change over time. Secondly, the measures of discrimination used were self-reported and therefore subject to recall bias. Thirdly, the questions were designed to measure age discrimination in the context of other sources of discrimination, and therefore may not be optimal. However, a more targeted measure may prime respondents to answer in a particular way, whereas in our study age discrimination was not the apparent focus of the items. Further, respondents were able to attribute more than one reason to their experiences of discrimination; therefore, it is not possible to establish for certain whether an individual situation was due to age discrimination or another type of discrimination. The decision to restrict the sample to White respondents only, to increase the comparability between the two study populations, makes it difficult for us to say how perceived age discrimination differs across racial groups. However, analyses not shown here did indicate that the same overall associations were found in both the United States and England. Finally, there may be factors that we have not captured here which may influence perceptions of age discrimination, for example, the effect of social networks and intergenerational closeness in both countries.

In summary, we found that levels of perceived age discrimination are significantly lower overall in the United States in comparison with England. While we cannot identify the specific reason for the observed U.S. advantage, we can surmise that that differing social and political circumstances in the two countries may have an important role to play. Since we measured perceived age discrimination, we cannot draw conclusions about levels of actual age discrimination. Nonetheless, the findings may be indicative of how older age is perceived in each country. Age discrimination is an important issue in both England and the United States and has the potential to affect a sizable proportion of society.

## Supplementary Material

Supplementary material can be found at: http://psychsocgerontology.oxfordjournals.org/

## Funding

I.R. is supported by an Impact PhD studentship from the International Longevity Centre–UK (ILC-UK) and University College London. A.S. is funded by the British Heart Foundation.

## Supplementary Material

Supplementary Data
