# Supplementary material for: Greater Perceived Age Discrimination in England than the United States: Results from HRS and ELSA
Source: J Gerontol B Psychol Sci Soc Sci. 2015 Jul 29;70(6):925–33. doi: 10.1093/geronb/gbv040 (PMC4600302; doi:10.1093/geronb/gbv040)
Supplement: Supplementary Data [file supp_70_6_925__index.html]

Greater Perceived Age Discrimination in England than the United States: Results from HRS and ELSA — Supplementary Data 

# Greater Perceived Age Discrimination in England than the United States: Results from HRS and ELSA

## Supplementary Data

Data files

- Supplementary Data - Supplementary Data
