# Supplementary material for: Greater Perceived Age Discrimination in England than the United States: Results from HRS and ELSA
Source: J Gerontol B Psychol Sci Soc Sci. 2015 Jul 29;70(6):925–33. doi: 10.1093/geronb/gbv040 (PMC4600302; doi:10.1093/geronb/gbv040)
Supplement: Supplementary Data [file supp_gbv040_HRS_ELSA_age_discrimination_Supplementary_Table_1_revision3.docx]

**Supplementary Table 1**. Proportion of respondents attributing discrimination to their age in different discriminatory situations in the USA and England

|  | Less Courtesy | | Medical Setting | | Less Clever | | Service Setting | | Harassed | |
| --- | --- | --- | --- | --- | --- | --- | --- | --- | --- | --- |
|  | **USA** | **England** | **USA** | **England** | **USA** | **England** | **USA** | **England** | **USA** | **England** |
| Age Discrimination | 14.8** | 18.2 | 9.2* | 10.3 | 12.9** | 11.1 | 7.9 | 8.8 | 2.7** | 4.5 |
| Age in years |  |  |  |  |  |  |  |  |  |  |
| 52-59 | 17.0 | 18.1 | 11.6 | 9.4 | 13.8* | 10.7 | 9.8 | 9.3 | 3.5* | 5.6 |
| 60-69 | 15.4** | 20.1 | 8.9* | 10.9 | 11.8 | 10.6 | 8.2 | 8.9 | 2.8** | 4.4 |
| 70-79 | 14.5* | 17.4 | 9.1 | 10.4 | 13.1 | 12.0 | 7.7 | 9.1 | 2.5** | 4.2 |
| Over 80 | 12.7 | 13.1 | 8.3 | 9.6 | 13.9 | 11.7 | 6.4 | 6.7 | 2.2 | 3.0 |
| Sex |  |  |  |  |  |  |  |  |  |  |
| Male | 15.3** | 21.1 | 10.2 | 10.9 | 12.3 | 11.6 | 8.3* | 10.2 | 3.2** | 6.0 |
| Female | 14.4 | 15.9 | 8.6* | 9.8 | 13.4** | 10.7 | 7.5 | 7.7 | 2.3* | 3.3 |
| Wealth |  |  |  |  |  |  |  |  |  |  |
| Lowest 1 | 17.7* | 21.5 | 10.9 | 12.4 | 17.0 | 15.9 | 9.9 | 10.0 | 4.1 | 5.7 |
| 2 | 17.1 | 19.8 | 9.7 | 9.8 | 16.6 | 13.7 | 8.3 | 9.9 | 3.5* | 5.8 |
| 3 | 15.4* | 19.2 | 9.3 | 10.1 | 14.4** | 10.7 | 8.8 | 9.7 | 3.1 | 3.4 |
| 4 | 13.0** | 18.2 | 9.8 | 10.1 | 11.2 | 9.9 | 8.0 | 8.8 | 2.1** | 4.9 |
| Highest 5 | 12.9 | 13.4 | 7.8 | 9.5 | 8.8 | 7.0 | 5.6 | 6.2 | 1.5** | 3.1 |
| Education |  |  |  |  |  |  |  |  |  |  |
| Low | 16.0 | 16.3 | 10.0 | 8.9 | 15.8* | 12.3 | 8.3 | 8.6 | 3.8 | 3.9 |
| Intermediate | 13.5** | 19.6 | 7.9* | 9.7 | 12.7 | 11.7 | 6.8** | 9.0 | 2.2** | 4.5 |
| High | 15.2** | 18.0 | 9.9* | 12.0 | 12.0* | 9.8 | 8.4 | 8.8 | 2.6** | 4.9 |
| Marital Status |  |  |  |  |  |  |  |  |  |  |
| Married | 14.3** | 18.1 | 9.3 | 9.9 | 12.1** | 10.0 | 7.9 | 8.9 | 2.5** | 4.4 |
| Single | 17.4 | 18.2 | 10.1 | 10.7 | 17.4 | 11.4 | 10.1 | 9.1 | 6.4 | 7.1 |
| Divorced or Separated | 17.1* | 21.7 | 8.8* | 12.5 | 15.4 | 15.4 | 7.4 | 10.1 | 3.6 | 5.0 |
| Widowed | 15.1 | 16.1 | 9.0 | 10.1 | 14.2 | 12.7 | 7.9 | 7.5 | 2.5 | 3.5 |
| Work Status |  |  |  |  |  |  |  |  |  |  |
| Retired | 14.5** | 18.1 | 9.2* | 10.9 | 12.8 | 11.8 | 7.7 | 8.7 | 2.3** | 4.1 |
| Employed | 16.0* | 19.0 | 9.5 | 8.8 | 12.8* | 10.1 | 8.8 | 8.9 | 3.7 | 5.2 |
| Other | 14.2 | 16.4 | 8.8 | 10.8 | 14.2 | 10.4 | 7.1 | 9.4 | 3.1 | 5.0 |

*Notes*: *statistically significant differences between USA and England at p<.05.

** statistically significant differences between USA and England at p<.01
